# Age-related differences in primary central nervous system lymphomas based on the SEER database

**DOI:** 10.3389/fmed.2025.1534956

**Published:** 2025-03-26

**Authors:** Tengjiao Lin, Ke Wang, Deyu Yang, Zhichao Li, Chuangjie Zheng, Xinrong Chen, Linzhu Zhai

**Affiliations:** ^1^Cancer Center, Departments of Radiation Oncology, The First Affiliated Hospital of Guangzhou University of Chinese Medicine, Guangzhou, China; ^2^Guangzhou University of Chinese Medicine, Guangzhou, China; ^3^Lingnan Medical Research Center, Guangzhou University of Chinese Medicine, Guangzhou, China

**Keywords:** primary central nervous system lymphoma, SEER, population study, treatment modality, nomogram

## Abstract

**Objective:**

The aim of this study was to compare prognostic factors and survival outcomes in patients with primary central nervous system lymphoma (PCNSL).

**Methods:**

The Surveillance, Epidemiology, and End Results (SEER) database was queried for patients diagnosed with PCNSL between 2000 and 2019.

**Results:**

Between 2000 and 2019, a total of 5,812 patients were diagnosed with PCNSL, of whom 2,175 (37%) were ≤ 60 years old and 3,637 (63%) were >60 years old. The younger patients had a higher rate of being diagnosed with stage I–II, a higher rate of receiving chemotherapy and radiotherapy, a similar rate of receiving surgery, and a longer survival time. The most common histological subtype among PCNSL patients was diffuse large B-cell lymphoma (PCNS-DLBCL). Younger PCNS-DLBCL patients who received surgery and chemotherapy exhibited significantly improved overall survival (OS) and disease-specific mortality (DSM) and that African American patients were associated with poorer OS and DSM. Older patients receiving surgery, chemotherapy, and radiotherapy exhibited significantly improved OS and DSM, male and higher Ann Arbor stage were associated with poorer OS and DSM. We created a nomogram for PCNS-DLBCL to predict OS, with a C-index of 0.6749 in the younger cohort and 0.6676 in the older cohort. In the combined therapy analysis, chemotherapy combined with surgical resection had better OS and DSM in all patients.

**Conclusions:**

The two age-stratified cohorts significantly differed in terms of OS and independent influences on OS and DSM. Our constructed nomogram exhibited high accuracy in predicting OS in PCNS-DLBCL patients.

## Introduction

Primary central nervous system lymphoma (PCNSL) is an aggressive extra-nodal non-Hodgkin's lymphoma that arises directly in the central nervous system (1). The clinical manifestations of PCNSL are usually non-specific and include headache, vomiting, focal neurological deficits, and generalized neurological deterioration (2). The most common histological subtype of PCNSL is diffuse large B-cell lymphoma (PCNS-DLBCL), which accounts for over 90% of cases (3). In terms of morbidity, the PCNSL data from national population-based studies in Western Europe, North America, and Asia converge, with annual incidence rates of 0.3–0.5/100,000 people. However, the incidence of PCNSL varies by age group and is highest in patients aged 60 years and older (4).

Over the past three decades, treatment for PCNSL patients has improved considerably. Novel drugs, including immune checkpoint inhibitors, immunomodulatory drugs, Bruton's tyrosine kinase inhibitors, and PI3K/AKT/mTOR inhibitors, and CAR-T cell therapies have achieved good results in several clinical trials. However, traditional modalities including chemotherapy, radiotherapy, and surgery still play important roles in the treatment of PCNSL (5–9). Compared to systemic lymphomas outside the central nervous system, the prognosis of PCNSL remains poor. In a population-based study by the French oculo-cerebral lymphoma network, PCNSL patients (*n* = 1,002) treated according to current national guidelines had a median overall survival (OS) of 25 months and a 5-year survival rate of 38% (10). Age is the main independent prognostic factor for PCNSL with better prognoses in younger patients: the 5-year progression-free survival rates were 61% and 28% in patients under or above the age of 60 years, respectively (11). This suggests that clinicians should consider the impact of age on prognosis when choosing the optimal treatment for PCNSL patients.

In a recent study, a large cohort of 5,166 PCNSL patients was evaluated based on the Surveillance, Epidemiology, and End Results (SEER) database, and it was reported that race, sex, age, marital status, surgical resection, and chemotherapy were independent prognostic factors for OS (12). In another study, the risk factors for non-cancer-specific survival in PCNSL patients were male sex, black race, unmarried status, and a lack of chemotherapy (13). However, these previous studies did not directly compare the differences in prognosis and treatment options between younger and older PCNSL patients. Therefore, we stratified a large cohort of PCNSL patients according to age (≤ 60 vs. > 60 years) and analyzed the impact of age on survival outcomes in PCNSL patients.

## Methods

### Data source

Patient data were obtained through the SEER database (version 8.4.1). Through the “Incidence-SEER Research Plus Data, 18 Registries, Nov 2021 Sub (2000–2019),” patients diagnosed with PCNSL between 2000 and 2019 were identified. The International Classification of Diseases for Oncology (ICD-O-3) histology codes (9590–9595, 9650–9699, and 9702–9729) were used for lymphoma with primary sites confined to the central nervous system identified by site-specific codes (C71.0–C71.9). Patients with missing information regarding age at diagnosis, race, sex, marital status, and survival status and incomplete follow-ups were excluded. Data collected included demographic and clinical parameters, including staging, pathology, treatment modality, and survival information. Patients stratified according to their age and clinical parameters were compared. Clinical information on patients in the SEER database is publicly available and anonymous, and therefore our study did not require ethics committee approval or patient consent.

### Statistical analyses

Statistical analyses of the data collected were performed using R software (R version 4.2.3). Patient and disease characteristics were stratified by age group (≤ 60 vs. > 60 years) at the time of PCNSL diagnosis and summarized using descriptive statistics. Kaplan–Meier curves were used to analyze OS, and multifactorial Cox analysis was used to analyze factors influencing OS and disease-specific mortality (DSM) in PCNS-DLBCL patients. Column line plots were constructed using the nomogram function for predicting 1-, 3-, and 5-year OS. DSM, concordance index (C-index), and receiver operating characteristic (ROC) were plotted to validate the reliability of the model. Kaplan–Meier analysis of the effect of treatment on OS and DSM was used to investigate the efficacy of different treatments on different populations. All the above analyses were stratified by age group, and statistical significance was considered at *P* < 0.05.

## Results

### Cohort characteristics

A total of 5,812 patients were diagnosed with PCNSL in the United States during 2000–2019, of whom 2,175 (37%) were ≤ 60 years old and 3,637 (63%) were >60 years old, hereafter referred to as “younger” and “older” patients, respectively (Table 1). Their median age at diagnosis was 49 years (interquartile range: 1–60) and 72 years (61–96), respectively. When comparing younger and older PCNSL patients, the majority was of white ancestry (71 vs. 85%, *P* < 0.001); however, there was an increased prevalence of African Americans in the younger cohort (16 vs. 3%, *P* < 0.001). When comparing the Ann Arbor stage at diagnosis between the two groups, the younger cohort had a higher rate of being diagnosed with stage I–II PCNSL (64 vs. 60%, *p* < 0.001). Furthermore, 64% of the population received chemotherapy, and the younger cohort exhibited higher rates of chemotherapy (68 vs. 63%, *P* < 0.001) and radiotherapy (34 vs. 26%, *P* < 0.001). Conversely, similar rates of surgery (*P* = 0.574) were observed in the younger and older cohorts.

**Table 1:** 2000–2019 onwards cohort characteristics, stratified by age group of primary central nervous system lymphoma patients.

	**Total**	** ≤ 60**	**>60**	***P*-value**
	**(*N* = 5,812)**	**(*N* = 2,175)**	**(*N* = 3,637)**	
**Age**				
Mean (SD)	62.9 (15.4)	46.7 (11.0)	72.6 (7.35)	
Median [Min, Max]	65.0 [1.0, 96.0]	49.0 [1.0, 60.0]	72.0 [61.0, 96.0]	
**Sex**				**< 0.001**
Female	2,754 (47)	877 (40)	1,877 (52)	
Male	3,058 (53)	1,298 (60)	1,760 (48)	
**Race**				**< 0.001**
White	4,637 (80)	1,553 (71)	3,084 (85)	
Black	440 (8)	334 (16)	106 (3)	
Others^a^	735 (12)	288 (13)	447 (12)	
**Marital status**				**< 0.001**
Single	1,217 (21)	848 (39)	369 (10)	
Married^b^	4,595 (79)	1,327 (61)	3,268 (90)	
**Ann Arbor stage**				**< 0.001**
Stage I	3,460 (59)	1,346 (62)	2,114 (58)	
Stage II	96 (2)	41 (2)	55 (2)	
Stage III	26 (0)	9 (0)	17 (0)	
Stage IV	678 (12)	288 (13)	390 (11)	
Unknown	1,552 (27)	491 (23)	1,061 (29)	
**Chemotherapy**				**< 0.001**
Performed	3,749 (64)	1,475 (68)	2,274 (63)	
No/unknown	2,063 (36)	700 (32)	1,363 (37)	
**Radiation**				**< 0.001**
Performed	1,678 (29)	730 (34)	948 (26)	
No/unknown	4,134 (71)	1,445 (66)	2,689 (74)	
**Surgery**				0.574
Performed	2,190 (38%)	809 (37)	1,381 (38)	
No/unknown	3,622 (62)	1,366 (63)	2,256 (62)	
**Histological subtypes**				**< 0.001**
DLBCL	4,458 (77)	1,579 (73)	2,879 (80)	
BL	34 (1)	17 (1)	17 (1)	
CLL/SLL	17 (0)	5 (0)	12 (0)	
FL	62 (1)	19 (1)	43 (1)	
HL	12 (0)	6 (0)	6 (0)	
LPL	23 (0)	9 (0)	14 (0)	
PTCL	85 (2)	52 (3)	33 (1)	
MCL	7 (0)	2 (0)	5 (0)	
MZL	63 (1)	31 (1)	32 (1)	
NK/T	4 (0)	3 (0)	1 (0)	
Lymphoid neoplasm	590 (10)	264 (12)	326 (9)	
Other	457 (8)	188 (9)	269 (8)	
**Cause of death (COD)**				**< 0.001**
Alive	1,623 (28)	631 (29)	659 (18)	
Dead of other causes^c^	748 (13)	412 (19)	669 (18)	
Dead of PCNSL	3,441 (59)	1,132 (52)	2,309 (64)	

^a^American Indian/AK Native, Asian/Pacific Islander.

^b^Included divorced/separated/widowed patients.

^c^Included attributable to causes other than this cancer/missing.

Histological subtypes: DLBCL, diffuse large B-cell lymphoma; BL, Burkitt's lymphoma; CLL/SLL, chronic lymphocytic leukemia/small lymphocytic lymphoma; FL, follicular lymphoma; HL, Hodgkin Lymphoma NOS; LPL, lymphoplasmacytic lymphoma; PTCL, peripheral Tcell lymphoma; MCL, Mantle-cell lymphoma; MZL, Marginal-zone lymphoma; NK/T, NK/T-cell lymph; Lymphoid neoplasm, Lymphoid neoplasm, NOS.

Bold values indicates *P* < 0.05 that the difference is significant.

### Survival outcomes

**Figure 1:**
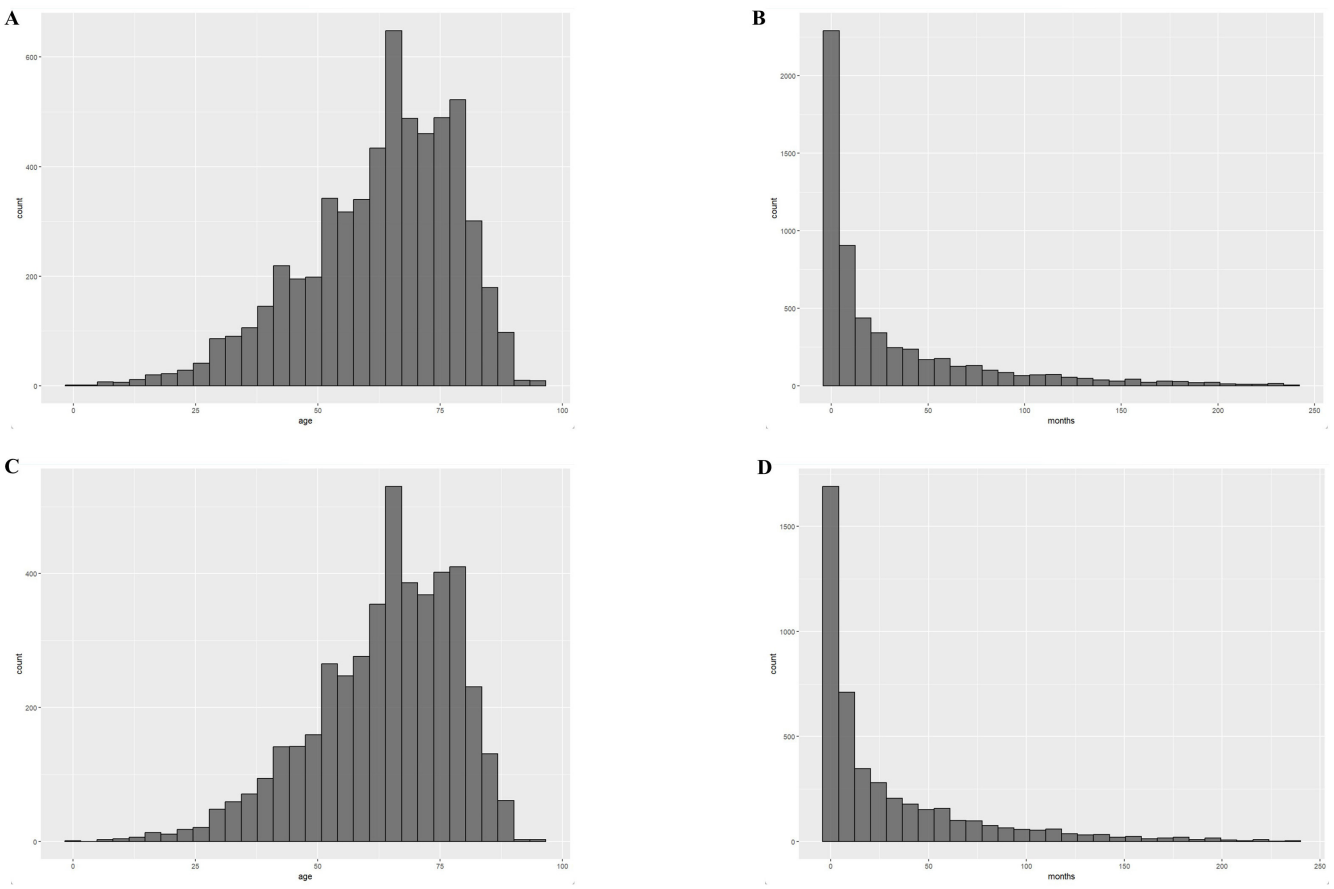
Distribution of age at diagnosis and survival time for the 2000–2019 cohort. **(A)** Histogram of age distribution of the PCNSL cohort; **(B)** Histogram of survival time distribution of the PCNSL cohort; **(C)** Histogram of age distribution of the PCNS-DLBCL cohort; **(D)** Histogram of survival time distribution of the PCNS-DLBCL cohort.

**Figure 2:**
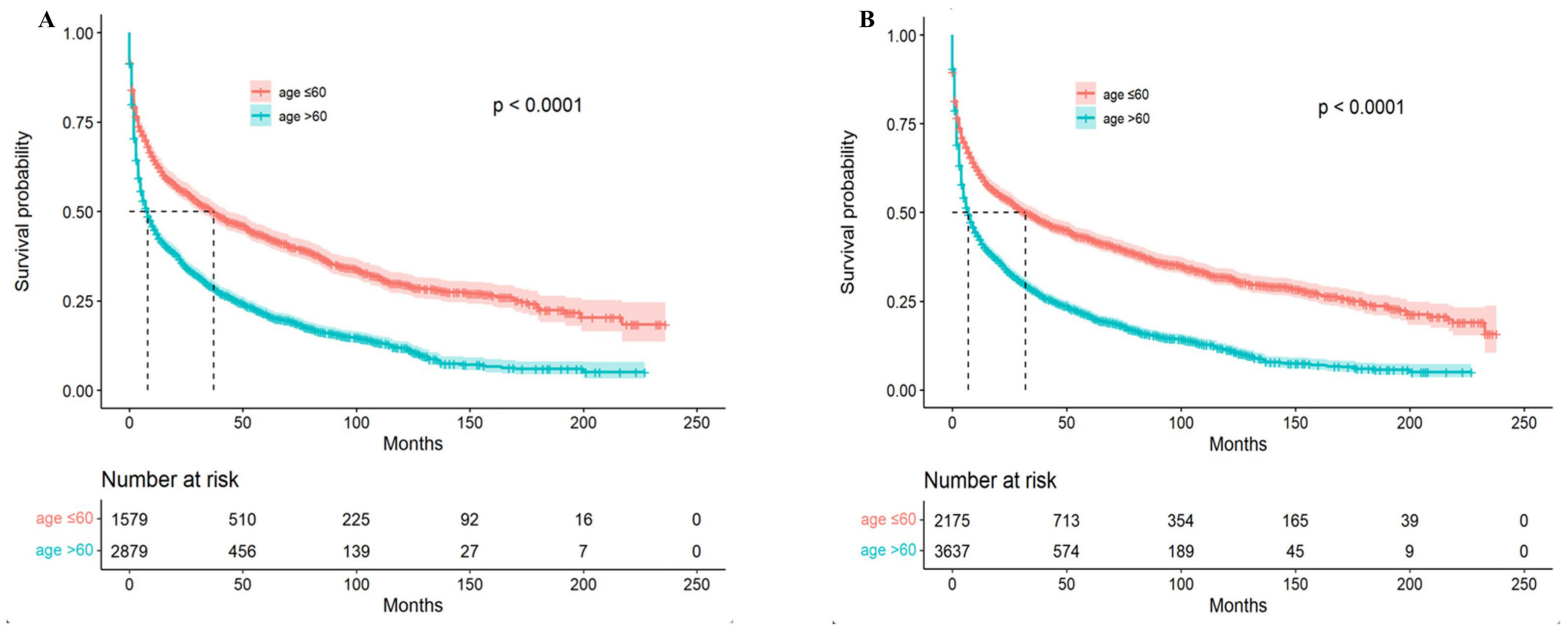
Overall survival (OS) of PCNSL patients stratified by age. **(A)** OS for PCNSL patients with a median survival time of 32 months (95% CI: 28–39) for patients ≤ 60 years of age and 7 months (95% CI: 6–8) for those >60 years of age, a significant difference, *P* < 0.0001; **(B)** OS for PCNS-DLBCL patients with a median survival time of 37 months (95% CI: 31–43) and 8 months (95% CI: 7–9) for >60 years, a significant difference, *P* < 0.0001.

The most common pathological subtype was DLBCL, which accounted for 77% of all cases (4,458/5,812) and was more common in older patients (80 vs. 73%, *P* < 0.001). Our analyses of the characteristics of the PCNS-DLBCL population revealed similar trends as those observed in PCNSL patients: the younger cohort had an increased prevalence of African Americans, higher rates of being diagnosed with stage I–II PCNSL, higher rates of receiving chemotherapy and radiotherapy, and similar rates of undergoing surgery (Table 2). The median age at diagnosis for all PCNSL patients was 65 years with a median survival time of 9 months, whereas the median age at diagnosis for DLBCL patients was 66 years with a median survival time of 10 months (Figure 1). Survival analyses revealed that compared to younger patients, older patients had higher death rates (64 vs. 52%, *P* < 0.001) and worse survival outcomes (*P* < 0.0001). The median survival time in younger patients was 32 months [95% confidence interval (CI): 28–39, *P* < 0.001], compared to 7 months (95% CI: 6–8, *P* < 0.001) in older patients. The results were similar in DLBCL patients, with a median survival time of 37 months (95% CI: 31–43, *P* < 0.001) in younger patients, compared to 8 months (95% CI: 7–9, *P* < 0.001) in older patients (Figure 2).

**Table 2:** 2000–2019 onwards cohort characteristics, stratified by age group among DLBCL patients.

	**Total**	** ≤ 60**	**>60**	***P*-value**
	**(*N* = 4,458)**	**(*N* = 1,579)**	**(*N* = 2,879)**	
**Age**				
Mean (SD)	63.6 (14.4)	47.8 (10.4)	72.3 (7.15)	
Median [Min, Max]	66.0 [1.00, 96.0]	50.0 [1.00, 60.0]	72.0 [61.0, 96.0]	
**Sex**				**< 0.001**
Female	2,133 (48)	646 (41)	1,487 (52)	
Male	2,325 (52)	933 (59)	1,392 (48)	
**Race**				**< 0.001**
White	3,606 (81)	1,170 (74)	2,436 (85)	
Black	262 (6)	186 (12)	76 (3)	
Others^a^	590 (13)	223 (14)	367 (12)	
**Marital status**				**< 0.001**
Single	856 (19)	552 (35)	304 (11)	
Married^b^	3,602 (81)	1,027 (65)	2,575 (89)	
**Ann Arbor stage**				**< 0.001**
Stage I	2,630 (59)	970 (61)	1,660 (58)	
Stage II	68 (1)	26 (2)	42 (1)	
Stage III	19 (1)	5 (0)	14 (1)	
Stage IV	500 (11)	197 (13)	303 (10)	
Unknown	1,241 (28)	381 (24)	860 (30)	
**Chemotherapy**				**< 0.001**
Performed	3,123 (70)	1,176 (75)	1,947 (68)	
No/unknown	1,335 (30)	403 (25)	932 (32)	
**Radiation**				**< 0.001**
Performed	1,248 (28)	513 (33)	735 (26)	
No/unknown	3,210 (72)	1,066 (67)	2,144 (74)	
**Surgery**				0.574
Performed	1,813 (41)	637 (40)	1,176 (41)	
No/unknown	2,645 (59)	942 (60)	1,703 (59)	
**Cause of death (COD)**				**< 0.001**
Alive	1,290 (29)	631 (40)	659 (23)	
Dead of other causes ^c^	529 (12)	131 (8)	398 (14)	
Dead of PCNSL	2,639 (59)	817 (52)	1,822 (63)	

^a^American Indian/AK Native, Asian/Pacific Islander.

^b^Included divorced/separated/widowed patients.

^c^Included attributable to causes other than this cancer/missing.

Bold values indicates *P* < 0.05 that the difference is significant.

DLBCL is the most common lymphoma subtype. Therefore, for the multivariate analysis, we constructed multivariate Cox proportional risk models for OS and DSM in DLBCL patients stratified by age. The 4,458 DLBCL patients were randomly divided into a training group (*n* = 3,121) and validation group (*n* = 1,337). Younger DLBCL patients undergoing surgery and chemotherapy exhibited significantly improved OS; male sex and black race were associated with poor prognosis, and Ann Arbor stage was not significantly associated with OS. Older DLBCL patients undergoing surgery, chemotherapy, and radiotherapy exhibited significantly improved OS; male sex and higher Ann Arbor staging were associated with poor prognosis (Table 3). We performed a further multivariate Cox regression analysis of DSM. A low incidence of DSM was found in younger patients receiving chemotherapy, radiotherapy, surgery; Ann Arbor stage I and black race were associated with poorer DSM. In older patients, undergoing surgery, chemotherapy, and radiotherapy and an earlier Ann Arbor stage were associated with better DSM; men had a higher incidence of DSM than women (Table 4).

**Table 3:** Multivariable Cox proportional hazards model for overall survival (OS) among DLBCL patients.

**Covariate**	≤ **60 years old**			>**60 years old**		
	**HR^a^**	**95% CI^a^**	***P*-value**	**HR^a^**	**95% CI^a^**	***P*-value**
Male	1.16	1.01, 1.33	**0.033**	1.14	1.05,1.24	**0.002**
Black race	1.25	1.03, 1.52	**0.021**	0.88	0.68,1.15	0.4
Chemotherapy yes	0.32	0.28, 0.37	**< 0.001**	0.3	0.27,0.33	**< 0.001**
Radiation yes	1.06	0.92, 1.21	0.4	0.82	0.74,0.90	**< 0.001**
Surgery yes	0.75	0.66, 0.86	**< 0.001**	0.76	0.70,0.83	**< 0.001**
Ann arbor stage I	1.1	0.91, 1.34	0.3	1.11	1.00, 1.23	0.054
Ann Arbor stage II	1.32	0.82, 2.15	0.3	1.08	0.76,1.54	0.7
Ann Arbor stage III	0.87	0.32, 2.38	0.8	1.92	1.13,3.27	**0.016**
Ann Arbor stage IV	1.25	0.98, 1.60	0.076	1.18	1.02,1.38	**0.031**

^a^HR, Hazard Ratio; CI, Confidence Interval.

Bold values indicates *P* < 0.05 that the difference is significant.

**Table 4:** Multivariable competing risks regression model for disease-specific mortality (DSM) among DLBCL patients.

**Covariate**	≤ **60 years old**			>**60 years old**		
	**HR^a^**	**95% CI^a^**	***P*-value**	**HR^a^**	**95% CI^a^**	***P*-value**
Male	0.97	0.84, 1.12	0.7	1.11	1.01, 1.22	**0.023**
Black race	1.58	1.28, 1.95	**< 0.001**	0.79	0.58, 1.06	0.12
Chemotherapy yes	0.37	0.31, 0.43	**< 0.001**	0.39	0.35, 0.43	**< 0.001**
Radiation yes	0.64	0.56, 0.74	**< 0.001**	0.67	0.60, 0.75	**< 0.001**
Surgery yes	0.86	0.74, 0.99	**0.039**	0.9	0.82, 0.99	**0.028**
Ann Arbor stage I	0.5	0.41, 0.62	**< 0.001**	0.62	0.56, 0.70	**< 0.001**
Ann Arbor stage II	1.22	0.71, 2.11	0.5	0.65	0.44, 0.96	**0.031**
Ann Arbor stage III	1.66	0.52, 5.28	0.4	1.49	0.77, 2.90	0.2
Ann Arbor stage IV	0.67	0.52, 0.88	**0.004**	1.32	0.82, 2.43	0.16

^a^HR, Hazard Ratio; CI, Confidence Interval.

Bold values indicates *P* < 0.05 that the difference is significant.

### Construction of the nomogram

**Figure 3:**
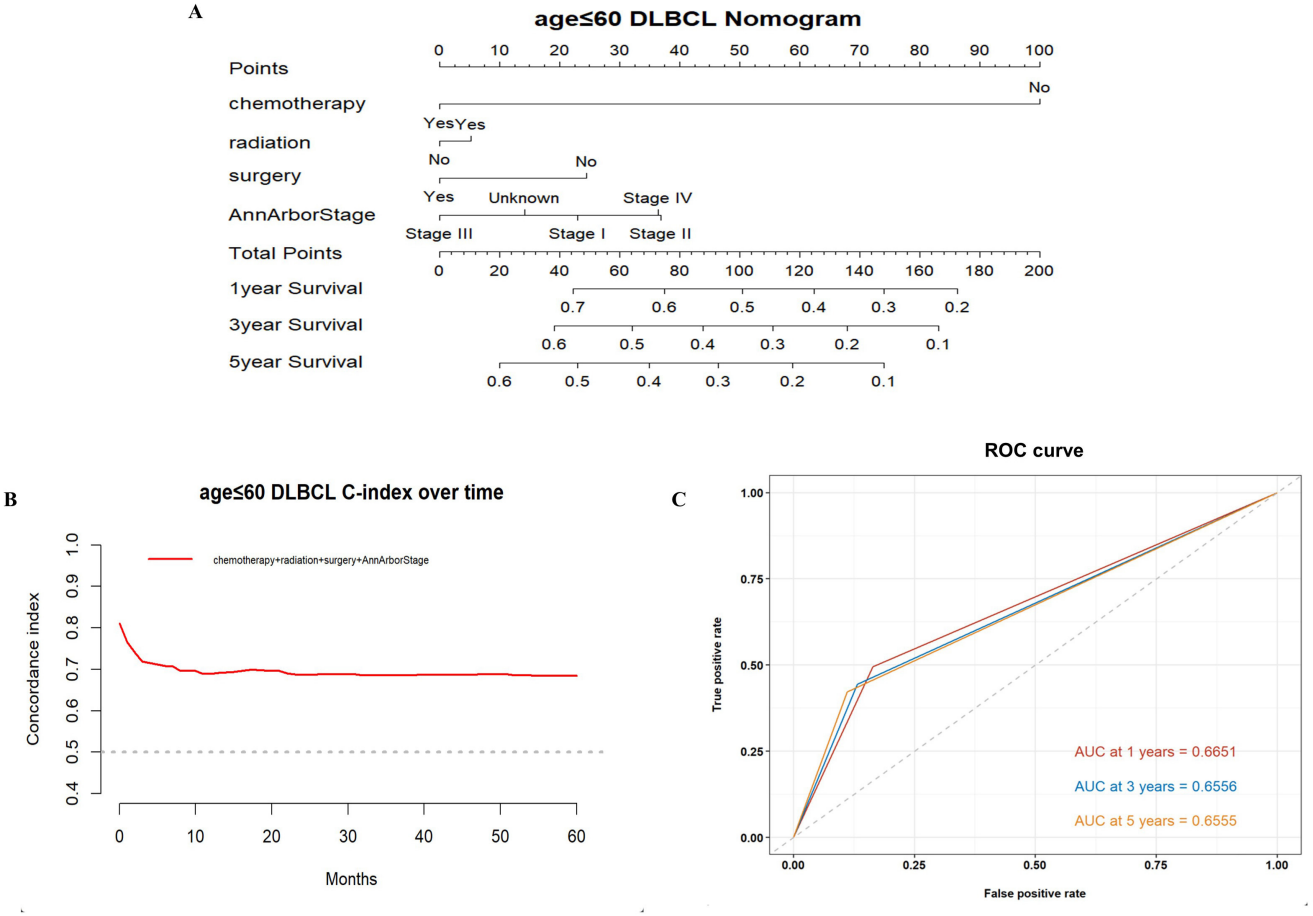
Survival nomogram, C-index, and ROC curves for patients ≤ 60 years of age with PCNS-DLBCL. **(A)** Prognostic nomogram integrating the independent prognostic factors for predicting OS; **(B)** C-index curves; **(C)** ROC curves for predicting patient survival at 1, 3, and 5 years.

**Figure 4:**
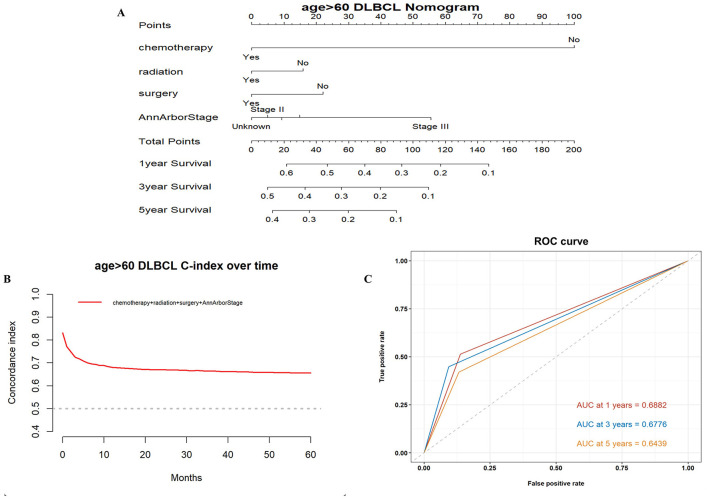
Survival nomogram, C-index, and ROC curves for patients >60 years of age with PCNS-DLBCL. **(A)** Prognostic nomogram integrating the independent prognostic factors for predicting OS; **(B)** C-index curves; **(C)** ROC curves for predicting patient survival at 1, 3, and 5 years.

Based on the results of these analyses, we created OS nomograms for 1, 3, and 5 years in PCNS-DLBCL patients (Figures 3, 4). We discriminated and calibrated the nomograms by internal validation cohorts. In the younger cohort, the C-index value of OS was 0.6749 (95% CI: 0.6641–0.6857), and the areas under the ROC curves for 1-, 3-, and 5-year survival were 0.6651, 0.6556, and 0.6555, respectively. In the older cohort, the C-index value of OS was 0.6676 (95% CI: 0.6595–0.6759), and the areas under the ROC curves for 1-, 3-, and 5-year survival were 0.6882, 0.6776, and 0.6439, respectively. These results suggest that columnar plots have good predictive value.

### Combination therapy strategy analysis

**Figure 5:**
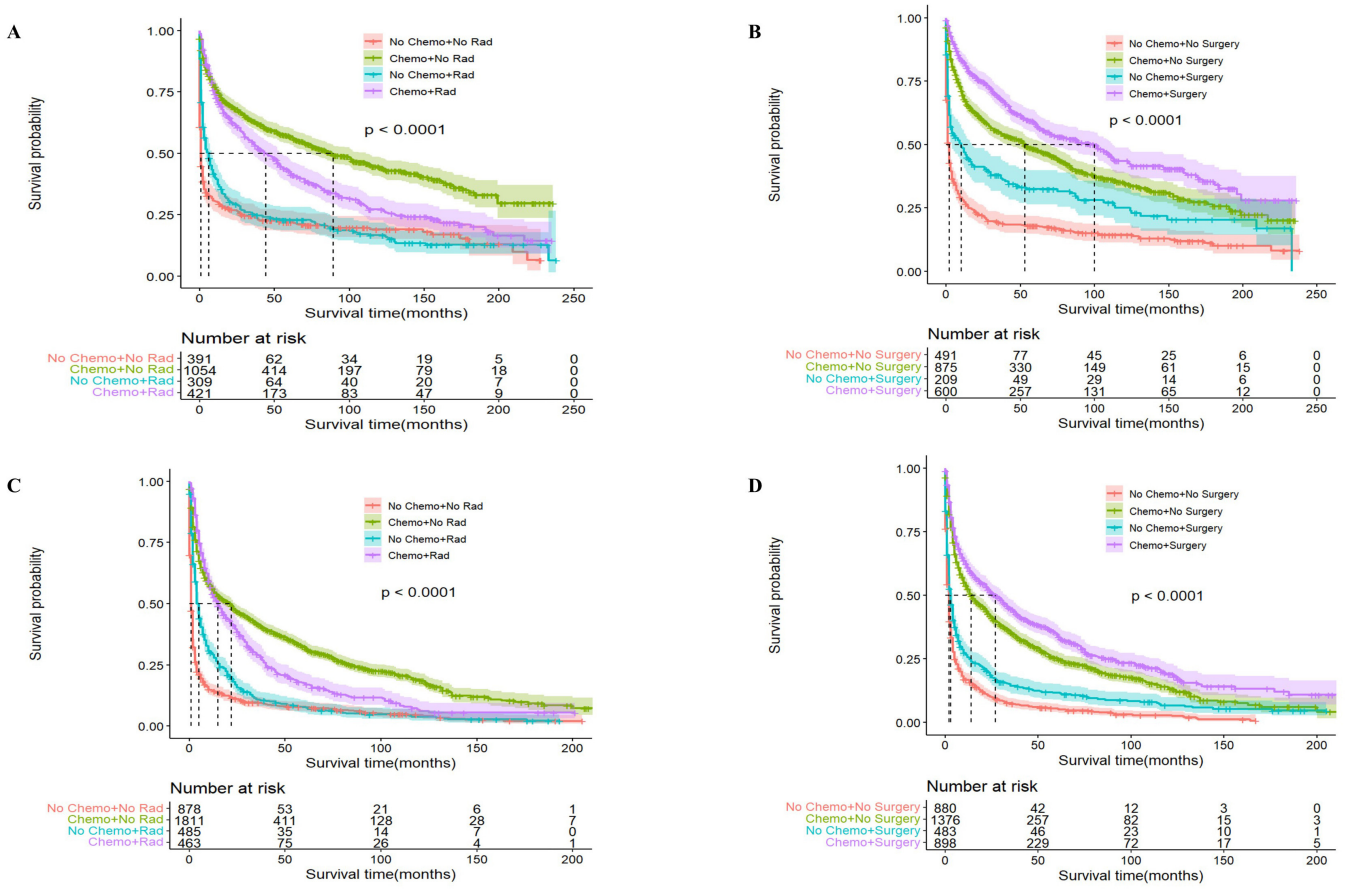
Analysis of the effect of combination therapy on OS in PCNSL patients by age stratification. For patients ≤ 60 years of age with PCNSL, chemotherapy combined with radiotherapy **(A)** and chemotherapy combined with surgery **(B)**; for patients >60 years of age with PCNSL, chemotherapy combined with radiotherapy **(C)** and chemotherapy combined with surgery **(D)**; surgery combined with chemotherapy was significantly associated with better OS, *P* < 0.0001.

**Figure 6:**
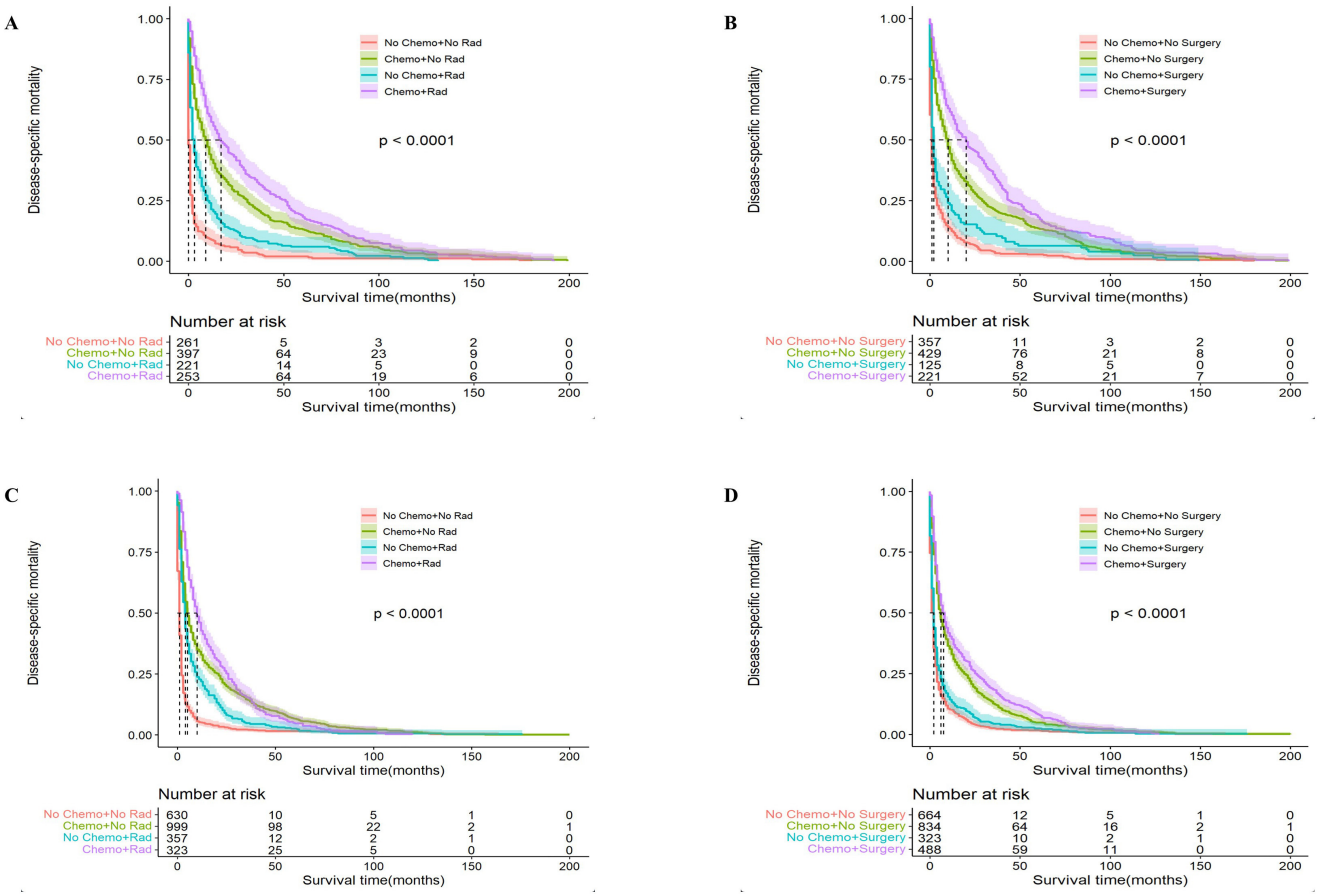
Analysis of the effect of combination therapy on Disease-Specific Mortality (DSM) in PCNSL patients by age stratification. For patients ≤ 60 years of age with PCNSL, chemotherapy combined with radiotherapy **(A)** and chemotherapy combined with surgery **(B)**; for patients >60 years of age with PCNSL, chemotherapy combined with radiotherapy **(C)** and chemotherapy combined with surgery **(D)**; surgery combined with chemotherapy was significantly associated with better DSM, *P* < 0.0001.

Next, we analyzed the effectiveness of combination therapy in PCNSL patients. In the younger cohort, chemotherapy combined with surgical resection had better OS with a median survival time of 100 months (95% CI: 70–112, *P* < 0.0001), whereas chemotherapy combined with radiotherapy resulted in worse OS with a median survival time of 44 months (95% CI: 36–54, *P* < 0.0001; Figure 5). The same trend was obtained in the older cohort. Chemotherapy combined with surgical resection had a better OS with a median survival time of 27 months (95% CI: 22–33, *P* < 0.0001), whereas radiotherapy combined with chemotherapy led to a worse OS with a median survival time of 10 months (95% CI: 9–12, *P* < 0.0001; Figure 6). Compared to monotherapy, chemotherapy combined with surgical resection and radiotherapy combined with chemotherapy have better DSM and are not affected by age.

## Discussion

PCNSL has a low incidence but is a highly aggressive non-Hodgkin lymphoma with poor prognosis. To date, most studies of PCNSL have been based on retrospective analyses of small samples. Fewer studies have assessed the prognosis of PCNSL patients by age stratification. This study included a total of 5,812 patients diagnosed with PCNSL in the United States during 2000–2019. The median age of all PCNSL patients at diagnosis was 65 years, a female-to-male ratio of 1:1.13. We divided PCNSL patients into a younger cohortand an older cohort. The results showed that younger patients had a better OS than older patients. The median survival time for younger patients was 32 months, whereas that for older patients was only 7 months. We obtained the same results in the subgroup of DLBCL patients, indicating a significant difference in prognosis between younger and older patients.

To date, histopathology remains the gold standard for the diagnosis of PCNSL. The conventional view is that surgical resection may lead to neurological damage and cannot be considered the preferred option (14). However, recent years have seen advances in neurosurgery, and it has thus been noted that surgical resection provides a better survival benefit to PCNSL patients. In a retrospective study of 70 patients, Wu et al. found that surgical resection resulted in longer OS than puncture biopsy (23.4 vs. 11.2 months) (15). Jelena et al. showed similar results in a retrospective study of 27 patients, with longer OS in patients who underwent surgical resection (16). In our study, a multifactorial Cox model was used to analyze OS and DSM in 4,458 PCNS-DLBCL patients, and the results showed that both younger and older patients undergoing surgical resection had longer OS and lower DSM. Puncture biopsy has the advantage of being less invasive than resection and has a shorter recovery time for the patient. However, in some patients with large occupying lesions or at risk of cerebral herniation at any time, surgical resection can rapidly reduce intracranial pressure and provide a survival benefit (17). Our study shows the advantage of surgical resection, and this advantage should be validated in a large prospective study.

High-dose-methotrexate-based chemotherapy regimens, which remain the standard of care for PCNSL systemic therapy, are unanimously recommended by multinational guidelines and have been proven effective with manageable side effects in numerous studies (18–20). In our study, 3,123 PCNS-DLBCL patients (70%) received chemotherapy. Multifactorial Cox analysis showed that chemotherapy was significantly associated with better OS and DSM in both younger and older patients. This suggests that although new drugs are emerging for the treatment of PCNSL patients, the role of chemotherapy in the treatment of PCNS-DLBCL patients should not be overlooked.

The role of whole brain radiation therapy (WBRT) in the treatment of PCNSL has been controversial. In one multicenter phase 3 trial, patients receiving WBRT had longer progression-free survival but no statistically significant difference in OS (21). In another study, there were no significant differences in progression-free survival and OS between controls and patients receiving WBRT (22). Additionally, WBRT has a high risk of neurotoxicity and is not considered to be the preferred consolidation treatment modality (23, 24). Based on our large population analysis, radiotherapy was associated with better DSM in both PCNS-DLBCL cohorts in this study. It is noteworthy that in the older cohort, radiotherapy significantly improved OS, which differs from the findings of Tang et al. (12). The possible reasons for this difference include that older people may be more likely to undergo stereotactic body radiation therapy or stereotactic ablative radiotherapy for radiation treatment with less side effects due to their age and medical condition, whereas younger people are more likely to undergo WBRT. In addition, our study shows that the elderly have shorter OS, and thus central nervous system toxicity caused by radiotherapy may not be fully manifested. Conversely, this side effect will be more obvious in younger patients with longer OS. Although older patients may benefit from radiation, this result from our study must be interpreted with caution.

We found that African Americans were associated with poorer OS and DSM in the older cohort. No studies have demonstrated a relationship between ethnicity and survival prognosis, and thus we speculate that this result may be related to the socioeconomic status of African Americans. In addition, we explored the impact of combination therapy on survival benefit in PCNSL patients of different ages, expecting to obtain the combination therapy modality that would provide the greatest benefit in patients of different ages. In both the younger and older cohorts, chemotherapy combined with surgical resection had better OS and DSM. Conversely, chemotherapy combined with radiotherapy resulted in poorer OS and was not superior to chemotherapy alone. This may be due to the occurrence of leukodystrophy after concurrent high-dose methotrexate chemoradiotherapy in the elderly and impaired cognitive function and delayed neurological toxicity caused by radiotherapy, as evidenced by the crossover of survival curves between chemotherapy combined with radiotherapy and chemotherapy alone in the older cohort. This suggests to clinicians that when choosing a combination therapy for PCNSL patients, chemotherapy combined with surgical resection provides a better survival benefit. By contrast, it is questionable whether the combination with radiotherapy provides any long-term benefits to patients. However, this conclusion needs to be further supported by prospective studies with large samples.

The nomogram is a valid and convenient statistical tool that incorporates variables affecting prognosis and can be used for the prediction of prognosis in a wide range of cancers (25, 26). This study incorporated independent prognostic factors, such as surgery, radiotherapy, chemotherapy, and Ann Arbor stage, to develop a nomogram to predict OS after 1, 3, and 5 years in PCNS-DLBCL patients. Its validity was evaluated using the C-index and ROC curve, which revealed that the prognostic model has good accuracy and predictability.

Our study also has some limitations. First, this is a retrospective study, and thus there is a degree of selection bias. Second, the data from the SEER database is limited, and thus details of the treatment, e.g., the specific chemotherapy regimen or radiation dose, are not available. Finally, due to the lack of multicenter clinical data, the predictive model we constructed could only be tested by internal validation. This may have contributed to any inaccuracies in the conclusions of this study. Further multicenter and multidisciplinary prospective clinical studies are needed to explore age-based prognostic stratification of PCNSL patients.

## Conclusions

In summary, a comprehensive analysis of PCNSL patients from 2000 to 2019 based on the SEER cancer database showed that patients ≤ 60 years of age had better OS. For PCNS-DLBCL patients ≤ 60 years of age, surgery, chemotherapy, race, sex, and Ann Arbor stage were independent factors influencing OS and DSM. For PCNS-DLBCL patients > 60 years of age, surgery, chemotherapy, radiotherapy, sex, and Ann Arbor stage were independent factors influencing OS and DSM. Despite the limitations of this study, nomograms based on these factors predicted clinical outcomes in PCNS-DLBCL patients with high accuracy and applicability. Additionally, based on the SEER database, chemotherapy combined with surgical resection resulted in better OS and DSM for PCNSL patients, whereas radiotherapy performed poorly in the long term. With further research into the molecular biology of PCNSL and the introduction of more targeted therapies, we believe that the prognosis of PCNSL patients will be further improved.

## Data Availability

Publicly available datasets were analyzed in this study. This data can be found at: http://seer.cancer.gov.
